# Cardiac Tamponade and Duodenal Hemorrhage Caused by Inappropriate Use of Dabigatran in a Patient With End-Stage Renal Failure: A Case Report

**DOI:** 10.7759/cureus.52521

**Published:** 2024-01-18

**Authors:** Marina Hayashida, Masataka Suzuki, Yosuke Nakata, Hiroko Kakita, Hiroshi Eizawa

**Affiliations:** 1 Nephrology, Kobe City Nishi-Kobe Medical Center, Kobe, JPN; 2 Cardiology, Kobe City Nishi-Kobe Medical Center, Kobe, JPN

**Keywords:** gastrointestinal bleeding, cardiac tamponade, end-stage renal failure, idarucizumab, dabigatran etexilate

## Abstract

A 72-year-old man with end-stage renal failure, receiving 220 mg of dabigatran for chronic atrial fibrillation, was admitted with generalized edema and shortness of breath. Cardiac tamponade caused by pericardial hemorrhage due to inappropriate dabigatran use was treated with pericardial drainage and idarucizumab. Although coagulability normalized, consecutive duodenal hemorrhages occurred, requiring arterial embolization for hemostasis. In cases of severely impaired renal function, the usual dose of idarucizumab may not be sufficient to reverse the effects of dabigatran. Therefore, we considered the need for repeated idarucizumab administration to prevent recurrent bleeding.

## Introduction

Dabigatran, a direct thrombin inhibitor, is the first direct oral anticoagulant (DOAC) to be introduced in Japan for the prevention of stroke and systemic embolism in patients with atrial fibrillation [1]. Dabigatran is prescribed as 150 mg twice daily or 110 mg twice daily, based on age (≥70 years), moderate renal impairment (creatinine clearance (CrCl) 30-50 mL/min), gastrointestinal bleeding, and concomitant medication (P-glycoprotein inhibitors). Dabigatran is contraindicated in patients with severe renal impairment (CrCl < 30 mL/min) [2]. However, the surveillance indicated occasional prescription of dabigatran to patients with CrCl < 30 mL/min, resulting in unexpected adverse bleeding events [3]. Idarucizumab, a monoclonal antibody, rapidly reverses dabigatran's anticoagulant activity for fatal bleeding or medical procedures [4]. However, the efficacy of idarucizumab in patients with end-stage renal failure is not well established.

Herein, we report a case of cardiac tamponade and duodenal hemorrhage caused by inappropriate use of dabigatran in a patient with end-stage renal failure. Furthermore, this case highlights the possibility of re-elevated dabigatran concentration even after idarucizumab administration and the need for repeated administration.

## Case presentation

A 72-year-old man with a history of chronic kidney disease (stage G5) due to nephrosclerosis was admitted with generalized edema and shortness of breath. He had undergone several surgeries for thyroid cancer. Radiotherapy was administered 90-120 days before the date of admission for cervical lymph node metastasis. Later discovery revealed that, for over five years, his family doctor had prescribed 220 mg of dabigatran to prevent cerebral embolism associated with chronic atrial fibrillation. Five days before admission, edema of the lower legs appeared. He visited our hospital and was prescribed a diuretic. However, the edema worsened and eventually led to shortness of breath. Therefore, he was urgently admitted to our hospital. On admission, his blood pressure was 98/66 mmHg, with an irregular pulse rate of 82 beats/min. He needed a 2-liter oxygen. On examination, the jugular veins were distended, and heart sounds were diminished and irregular. Bilateral coarse crackles were audible in the lower lungs. The patient presented with indurated edema of the extremities. He had a history of chronic kidney disease, hypertension, dyslipidemia, hyperuricemia, and chronic atrial fibrillation. His daily medications included sodium zirconium cyclosilicate (5 g), sodium bicarbonate (3 g), alfacalcidol (0.25 µg), precipitated calcium carbonate (3 g), ferrous citrate (50 mg), levothyroxine sodium (175 µg), losartan (100 mg), amlodipine (10 mg), furosemide (40 mg), and dabigatran (220 mg).

**Figure 1:**
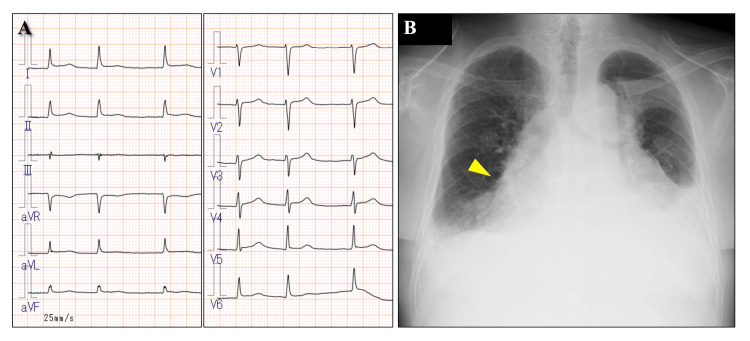
Electrocardiogram and chest radiograph (A) Electrocardiogram showed atrial fibrillation with low potentials. (B) Chest radiograph showed cardiac enlargement (cardiothoracic ratio 68%).

**Figure 2:**
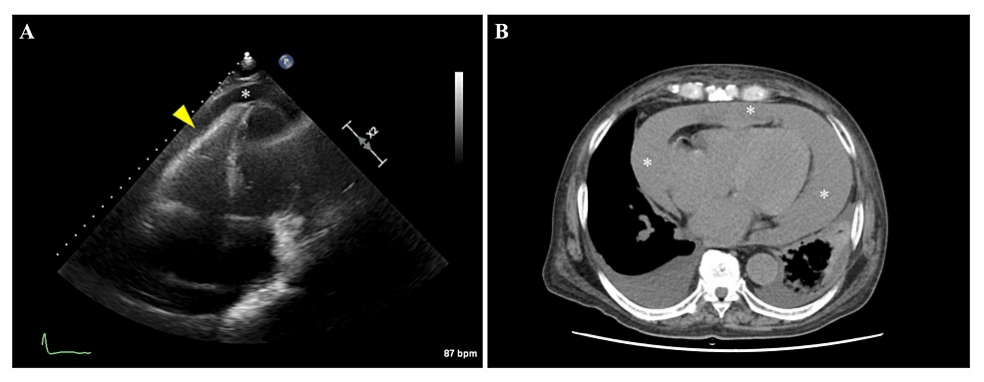
Transthoracic echocardiography and computed tomography (A) A four-chamber image of transthoracic echocardiography revealed a large pericardial effusion (asterisk) and compression of the right ventricle in early diastole (yellow triangle). (B) A computed tomography (CT) scan revealed significant pericardial effusion (asterisks). The CT value of pericardial effusion was 35 Hounsfield units, suspected to be hematogenous, whereas that of pleural effusion was 10 Hounsfield units.

Blood tests showed anemia (hemoglobin 9.0 mg/dL), decreased renal function (Cr 8.11 mg/dL, estimated glomerular filtration rate 5.8 mL/min/1.73 m^2^, CrCl 9.4 mL/min), and marked coagulation abnormalities (prothrombin time -international normalized ratio 2.3, activated partial thromboplastin time 95.4 s) (Table 1). The electrocardiogram showed known atrial fibrillation at a rate of 74 and low potentials, whereas no ST-T changes were observed (Figure 1A). Chest radiography revealed cardiac enlargement (Figure 1B). Transthoracic echocardiography revealed a large pericardial effusion and compression of the right ventricle in early diastole, consistent with cardiac tamponade (Figure 2A). Chest computed tomography (CT) revealed a substantial pericardial effusion, suggesting a hematogenous origin due to the elevated CT value (Figure 2B).

**Table 1:** Laboratory data on admission

Complete blood count		
White blood cells	8000	/µL
Red blood cells	333×10^4^	/µL
Hemoglobin	9.0	g/dL
Hematocrit	27.9	%
Platelets	38.4×10^4^	/µL
Neutrophil	89.3	%
Lymphocyte	3.0	%
Monocyte	7.1	%
Eosinophil	0.3	%
Basophil	0.3	%
Coagulation test		
Prothrombin time-international normalized ratio	2.3	
Activated partial thromboplastin time	95.4	s
D-dimer	5.9	µg/mL
Biochemistry		
Aspartate aminotransferase	14	IU/L
Alanine aminotransferase	21	IU/L
Lactate dehydrogenase	218	IU/L
Alkaline phosphatase	129	IU/L
Creatine kinase	80	IU/L
Total bilirubin	0.2	mg/dL
Total protein	6.9	g/dL
Albumin	2.7	g/dL
Urea nitrogen	128	mg/dL
Creatinine	8.1	mg/dL
Estimated glomerular filtration rate	5.8	mL/min/1.73m^2^
Sodium	132	mEq/L
Potassium	4.1	mEq/L
Chloride	92	mEq/L
Calcium	8.1	mg/dL
C-reactive protein	10.7	mg/dL
Glucose	233	mg/dL
Brain natriuretic peptide	88.3	pg/mL
High sensitivity myocardial troponin I	17.9	pg/mL
Thyroid stimulating hormone	3.225	mIU/L
Free T4	0.82	ng/dL
Anti-nuclear antibody	<40	times

Cardiac tamponade was considered due to massive bloody pericardial effusion, and urgent pericardial drainage was performed, which yielded 1.5 L of grossly bloody fluid. The pericardial fluid showed hematogenous characteristics, with no evidence of malignant cells and a negative bacterial culture (Table 2). Although radiation therapy was performed for thyroid cancer most recently, the mediastinum, including the pericardium, was out of the radiotherapy field. Coronary angiography revealed no evidence of coronary artery stenosis, occlusion, or contrast extravasation from the coronary arteries or veins. CT findings were not suggestive of aortic dissection. Blood tests were negative for endocrine and autoimmune diseases, with no history of trauma. Therefore, we diagnosed pericardial hemorrhage due to inappropriate use of dabigatran in a patient with end-stage renal failure. Dabigatran was discontinued after hospitalization, and 5 g of idarucizumab was administered to antagonize dabigatran.

**Table 2:** Laboratory data of the pericardial fluid

Data		
White blood cells	4,900	/µL
Red blood cells	298×10^4^	/µL
Hemoglobin	8.2	g/dL
Hematocrit	29.2	%
Platelets	3.1×10^4^	/µL
Neutrophil	93.3	%
Lymphocyte	3.9	%
Monocyte	2.0	%
Eosinophil	0.6	%
Basophil	0.2	%
pH	7.05	
Glucose	54	mg/dL
Total protein	5.5	g/dL
Lactate dehydrogenase	1051	IU/L
Adenosine deaminase	26.0	IU/L
Culture	No growth	
Cytology	﻿No malignant cells	

**Figure 3:**
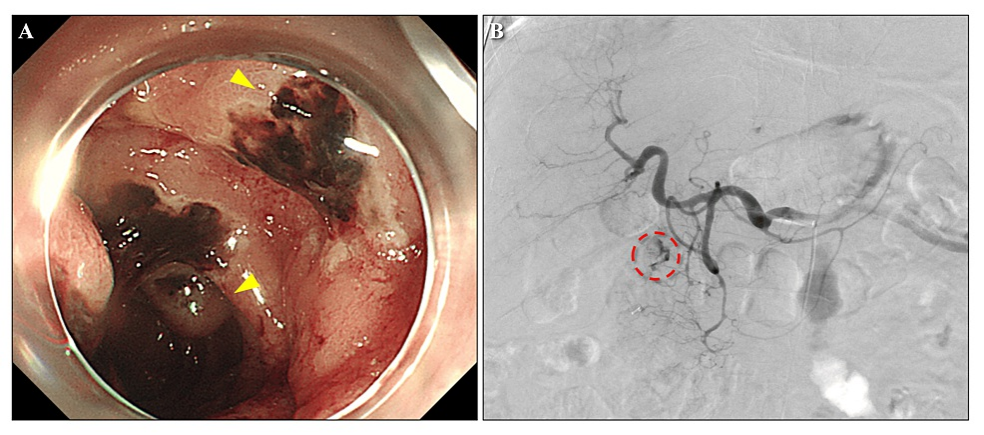
Gastrointestinal endoscopy and angiography (A) A gastrointestinal endoscopy revealed duodenal hemorrhage from acute duodenal mucosal lesions (yellow triangles). (B) An angiography showed extravasation of contrast medium from the anterior superior pancreaticoduodenal artery (red dashed circle). The bleeding was stopped using arterial embolization.

**Figure 4:**
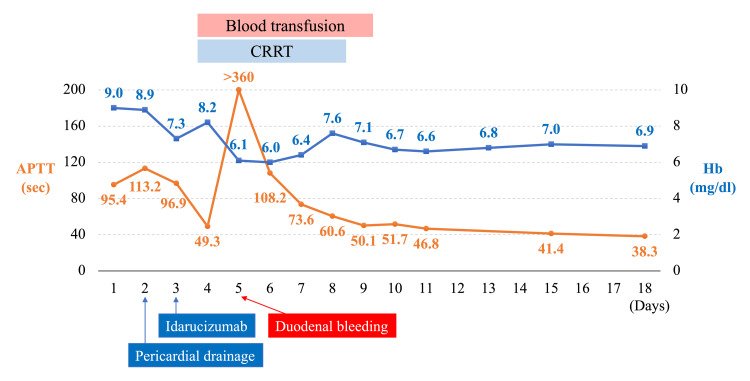
Patient’s clinical course After the administration of idarucizumab, the APTT was normalized; however, duodenal bleeding occurred with a remarkable re-elevation of the APTT. The APTT gradually decreased after the bleeding was stopped. APTT, activated partial thromboplastin time; CRRT, continuous renal replacement therapy; Hb, hemoglobin.

After receiving idarucizumab, coagulability normalized, and the bloody pericardial effusion resolved. However, the patient developed hypotension and became anuric on the fourth day. Norepinephrine and dobutamine were administered, and continuous renal replacement therapy (CRRT) was initiated using nafamostat mesylate as the anticoagulant. On the fifth day, significant melena, marked coagulation abnormality, and hypotension were observed. Massive blood transfusion and emergent gastrointestinal endoscopy were performed, uncovering hemorrhage from acute duodenal mucosal lesions in the descending part of the duodenum; however, stopping the bleeding via endoscopy proved challenging (Figure 3A). Interventional radiology was performed, and hemostasis was achieved through arterial embolization of bleeding from the anterior superior pancreaticoduodenal artery (Figure 3B). The patient’s general condition improved, and vasopressor administration was terminated. Urine output gradually increased, and the patient was weaned off dialysis. Coagulation abnormalities improved, and pericardial effusion or duodenal hemorrhage did not recur. The patient was discharged on the 23^rd^ day (Figure 4). The patient was followed up for chronic atrial fibrillation without anticoagulation therapy.

## Discussion

We encountered a case of hemorrhagic cardiac tamponade in a patient receiving dabigatran despite having poor renal function. The bloody pericardial effusion was treated with pericardial drainage and idarucizumab; however, a duodenal hemorrhage occurred consecutively.

Various conditions can cause pericardial effusion; however, the most common causes of hemorrhagic pericardial effusion are of medical origin, malignancy, myocardial infarction, and idiopathic causes. Trauma, aortic dissection, uremia, infection, collagen diseases, coronary artery dissection, and coronary aneurysms have also been reported [5]. Hematological disorders and bleeding predisposition, as well as anticoagulant and antiplatelet medications, are potential causes of bleeding. Particularly, bloody pericardial effusions due to DOAC have been reported [6-8]. In this case, the absence of malignant cells in the pericardial fluid, the lack of growth in the culture, and the absence of elevated adenosine deaminase ruled out malignancy, infection, or tuberculosis. No history of trauma existed, and the results from CT and coronary angiography eliminated aortic or coronary artery diseases. The patient had a predisposition to bleeding due to dabigatran despite poor renal function. Given this background, consider the possibility that the bleeding originated as a pericardial hemorrhage, and the hemostatic mechanism might not have kept pace, leading to the spread of bleeding within the pericardium and resulting in cardiac tamponade. The bloody pericardial effusion was successfully treated using pericardial drainage and idarucizumab.

Idarucizumab is a monoclonal antibody used as a reversal agent for dabigatran [4]. Once the patient’s coagulability normalized after idarucizumab administration and the pericardial hemorrhage resolved, the coagulation abnormality flared up, and fatal duodenal bleeding occurred approximately 48 h later. ﻿Based on plasma concentration data from the RE-LY trial, a 5-g dose of idarucizumab can reverse the total body load of dabigatran in 99% of patients with moderate renal dysfunction [9]. However, according to a subanalysis of the RE-VERSE AD trial, re-elevation of dabigatran levels within 12-24 h after idarucizumab administration is more common in patients with severe renal impairment (CrCl <30 mL/min) [10, 11]. A retrospective study indicated that dabigatran rebound is observed after idarucizumab reversal in patients with dabigatran concentrations >264 ng/mL [12]. Moreover, several case reports document exceptionally high blood concentration levels of dabigatran in patients with end-stage renal failure. These levels cannot be reversed even with regular doses of idarucizumab, necessitating repeated administration to stop recurrent bleeding [13-16]. The CrCl level in this patient significantly decreased to 9.4 mL/min on admission. Although the patient's blood dabigatran concentration was not measured, a possibility exists that the usual dose of idarucizumab was insufficient to counteract the dabigatran, resulting in the reappearance of coagulation abnormalities. We had to consider re-administration of idarucizumab in patients with severely impaired renal function. Unfortunately, in this case, a proton pump inhibitor was not administered to prevent stress ulcers. The stress from cardiac tamponade after hospitalization might cause acute mucosal lesions in the duodenum, leading to duodenal bleeding. Moreover, using anticoagulants, especially nafamostat mesylate, during CRRT may have contributed to coagulation abnormalities. Although not statistically significant, gastrointestinal bleeding and blood transfusions were reported to be increased in the nafamostat mesylate group compared to the no-anticoagulation group when CRRT was performed in patients at a high risk of bleeding [17]. Postulation attributes the duodenal hemorrhage to factors like incomplete dabigatran reversal, absence of prophylaxis for stress ulcers, and the use of anticoagulants during CRRT.

A survey on the use of DOAC found that 43% of patients with atrial fibrillation and renal impairment were potentially overdosed, resulting in a hazard ratio of >2 for major bleeding [18]. However, reports suggest that underdosed DOACs do not result in significant outcome differences compared to standard dosages [19]. Medical practitioners tend to administer lower doses of DOAC to the elderly or patients with renal impairment [20]. Whether underdosing improves the prognosis is debatable; however, overdosing can lead to fatal bleeding events, as in this case, and should be avoided. Regrettably, we did not have a complete understanding of the patient’s medication history, considering the severely impaired renal function and administration of dabigatran, which potentially led to a severe coagulation disorder. The incident underscores the need for sharing information between our team and the primary care physician regarding prescription details and blood test results, which could have prevented this serious bleeding event.

## Conclusions

A patient with severely impaired renal function was continuously prescribed dabigatran, which resulted in prolonged coagulation abnormalities and two life-threatening bleeding events. For the pericardial bleeding, coagulability was normalized by pericardial drainage and idarucizumab administration. However, fatal duodenal bleeding occurred later owing to multiple factors, such as incomplete dabigatran reversal, lack of prophylaxis for stress ulcers, and use of anticoagulants during CRRT. Additional idarucizumab administration may have prevented subsequent bleeding. In the future, when prescribing drugs dependent on renal excretion, regularly evaluate the patient's renal function to adjust medications as needed. Exercise caution to prevent overdosing and ensure communication of treatment details with family physicians while reviewing oral medications.
